# Supplementary material for: Bell correlations between spatially separated pairs of atoms
Source: Nat Commun. 2019 Oct 1;10:4447. doi: 10.1038/s41467-019-12192-8 (PMC6773866; doi:10.1038/s41467-019-12192-8)
Supplement: Supplementary file 1 — Supplementary information [file 41467_2019_12192_MOESM1_ESM.pdf]

Supplementary Information for

Bell correlations between spatially separated pairs of atoms

D. K. Shin, B. M. Henson, S. S. Hodgman, T. Wasak, J. Chwedeńczuk, and A. G. Truscott

SUPPLEMENTARY NOTE 1: DERIVATION OF NONLOCALITY CRITERIA

Amplitude criterion for entanglement

The correlator defined in Main Eq. 4, assuming local rotations around the y-axis by a common angle, can be written as follows

$$\mathcal{B}(\theta) = \frac{1}{2}[C_{xx} + C_{zz} + (C_{xx} - C_{zz})\cos 2\theta + (C_{xz} + C_{zx})\sin 2\theta], \tag{1}$$

where

$$C_{ij} = \left\langle \hat{\sigma}_i^{(A)} \hat{\sigma}_j^{(B)} \right\rangle \tag{2}$$

and $i, j = x, z$. The minimum and the maximum of $\mathcal{B}(\theta)$ with respect to θ yield $\mathcal{A}$ — the amplitude of oscillations, namely

$$\frac{1}{2}\left[C_{xx} + C_{zz} - \mathcal{A}\right] \leqslant \mathcal{B}(\theta) \leqslant \frac{1}{2}\left[C_{xx} + C_{zz} + \mathcal{A}\right], \tag{3}$$

where

$$\mathcal{A} = \sqrt{(C_{xx} - C_{zz})^2 + (C_{xz} + C_{zx})^2}. \tag{4}$$

If the two qubits form a separable (i.e., non-entangled) state, their composite density matrix reads

$$\hat{\varrho} = \int d\lambda\, p(\lambda) \hat{\varrho}_\lambda^{(A)} \otimes \hat{\varrho}_\lambda^{(B)}, \tag{5}$$

where $p(\lambda)$ is some probability distribution of a variable λ and $\hat{\varrho}^{(i)}(\lambda)$ are the single-qubit density matrices ($i = A, B$). These single-particle matrices are given by

$$\hat{\varrho}_\lambda^{(A)} = \frac{1}{2}(\hat{\mathbb{1}}^{(A)} + \vec{a}(\lambda)\hat{\vec{\sigma}}^{(A)}), \qquad \hat{\varrho}_\lambda^{(B)} = \frac{1}{2}(\hat{\mathbb{1}}^{(B)} + \vec{b}(\lambda)\hat{\vec{\sigma}}^{(B)}). \tag{6}$$

Here $\hat{\vec{\sigma}}^{(A/B)}$ is a vector of Pauli matrices for the qubit A/B and $\vec{a}(\lambda)$ and $\vec{b}(\lambda)$ are the corresponding Bloch vectors ($|\vec{a}(\lambda)| = 1$ for pure and $|\vec{a}(\lambda)| < 1$ for mixed states for A and analogously for B).

Using the separable state (Supplementary Eq. 5) this takes the form

$$C_{ij} = \mathrm{tr}\left\{\hat{\varrho}\hat{\sigma}_i^{(A)}\hat{\sigma}_j^{(B)}\right\} = \int d\lambda\, p(\lambda) a_i(\lambda) b_j(\lambda). \tag{7}$$

Note that the components of the Bloch vector lying in the zx-plane can be parameterised as follows

$$\vec{a}(\lambda) = \alpha(\lambda)\begin{pmatrix}\cos\phi_a^{(\lambda)} \\ \sin\phi_a^{(\lambda)}\end{pmatrix}, \quad \vec{b}(\lambda) = \beta(\lambda)\begin{pmatrix}\cos\phi_b^{(\lambda)} \\ \sin\phi_b^{(\lambda)}\end{pmatrix}, \tag{8}$$

where $\alpha(\lambda) \leqslant 1$ and $\beta(\lambda) \leqslant 1$. With this parameterisation, the amplitude $\mathcal{A}$ is equal to

$$\begin{aligned}\mathcal{A}^2 &= (C_{zz} - C_{xx})^2 + (C_{zx} + C_{xz})^2 \\ &= \langle\alpha\beta(\sin\phi_a \sin\phi_b - \cos\phi_a\cos\phi_b)\rangle^2 \\ &\quad + \langle\alpha\beta(\cos\phi_a\sin\phi_b + \sin\phi_a\cos\phi_b)\rangle^2 \\ &= \langle\alpha\beta\cos(\phi_a + \phi_b)\rangle^2 + \langle\alpha\beta\sin(\phi_a + \phi_b)\rangle^2.\end{aligned} \tag{9}$$

Using the Cauchy-Schwarz inequality

$$\langle\alpha\beta\cos(\phi_a + \phi_b)\rangle^2 \leqslant \left\langle(\alpha\beta)^2\right\rangle\left\langle\cos^2(\phi_a + \phi_b)\right\rangle \tag{10}$$

$$\langle\alpha\beta\sin(\phi_a + \phi_b)\rangle^2 \leqslant \left\langle(\alpha\beta)^2\right\rangle\left\langle\sin^2(\phi_a + \phi_b)\right\rangle \tag{11}$$

we obtain

$$\begin{aligned}\mathcal{A}^2 &\leqslant \left\langle(\alpha\beta)^2\right\rangle\left(\left\langle\cos^2(\phi_a+\phi_b)\right\rangle+\left\langle\sin^2(\phi_a+\phi_b)\right\rangle\right)\\ &=\left\langle(\alpha\beta)^2\right\rangle\left(\left\langle\cos^2(\phi_a+\phi_b)+\sin^2(\phi_a+\phi_b)\right\rangle\right)\\ &=\left\langle(\alpha\beta)^2\right\rangle\leqslant 1.\end{aligned} \tag{12}$$

Therefore $\mathcal{A} > 1$ implies entanglement between the qubits, which was used in Main Eq. 5.

Nonlocality

In our setup, the spin-rotation beams are much larger than the halo size at this point, thus all atoms in the halo are rotated by the same angle θ and we have access only to the diagonal part of E, as $\mathcal{B}(\theta) = E(\theta,\theta)$. An extension to implement independent rotations in each atom of the pairs would be experimentally possible, but is beyond the scope of this current work.

Still, using Main Eq. 4 we can test a wide range of LHV theories. These also take binary outcomes in A and B (i.e., $\uparrow$ / $\downarrow$), but assume that, on average, the results in one of the subsystems (either A or B) behave like components of a vector, while making no assumptions about the other part. To restrict our system to such an LHV theory, we analyse the properties of rotations in A and B in a dedicated series of experiments, with the results shown in Main Fig. 3 and Supplementary Figure 2 demonstrating that they do indeed rotate as vectors.

Consider two subsystems, where quantities A and B are measured. The joint probability for observing A and B fulfils the postulates of local realism if

$$P(A,B) = \sum_\lambda p(\lambda)P(A|\lambda)P(B|\lambda), \tag{13}$$

where $P(A|\lambda)$ or $P(A|\lambda)$ are the conditional probabilities for observing A or B given some value of a hidden variable λ, governed by the probability distribution λ. The conditional probability for observing B given some result A is

$$\begin{aligned}P(B|A) &= \frac{P(A,B)}{P(A)}\\ &=\sum_\lambda \frac{p(\lambda)P(A|\lambda)}{P(A)}P(B|\lambda)\\ &=\sum_\lambda P(\lambda|A)P(B|\lambda).\end{aligned} \tag{14}$$

Now, imagine that given the quantity measured in A, labelled as J_i^A, can have binary outcomes for two local settings $i = x, z$, i.e., $J_x^A = \pm 1$ and $J_z^A = \pm 1$. The two quantities J_i^B measured in B are, on average, assumed to be components of a vector of length 1, which in particular implies

$$-1 \leqslant \left\langle J_i^B \right\rangle \leqslant 1. \tag{15}$$

Note that we do not specify how this average is calculated, and the outcomes in B can be binary as well.

The average outcome in B, say in the x direction, given the result in A is

$$\begin{aligned}\left\langle J_x^B \right\rangle_A &= \sum_B P(B|A)J_x^B\\ &=\sum_\lambda P(\lambda|A)\sum_B P(B|\lambda)J_x^B,\end{aligned} \tag{16}$$

where

$$-J_\lambda^B \leqslant \sum_B P_Q(B|\lambda)J_x^B \leqslant J_\lambda^B, \tag{17}$$

and J_λ^B is the length of the vector in B given the value of λ. Thus the upper bound reads

$$\left\langle J_x^B \right\rangle_A = \sum_B P(B|A)J_x^B \leqslant \sum_\lambda P(\lambda|A)J_\lambda^B = J_A^B. \tag{18}$$

We take two orthogonal directions in the zx-plane, $\frac{1}{\sqrt{2}}(J_1^B - J_2^B)$, and according to the above argument we obtain

$$-J_A^B \leqslant \frac{\langle J_1^B \rangle_A - \langle J_2^B \rangle_A}{\sqrt{2}} \leqslant J_A^B. \tag{19}$$

Now, we modify the inequality (Supplementary Eq. 19) by multiplying the two averages by the corresponding results in A. Using that outcomes in A are binary, we obtain

$$-J_A^B \leqslant \frac{J_1^A \langle J_1^B \rangle_A - J_2^A \langle J_2^B \rangle_A}{\sqrt{2}} \leqslant J_A^B. \tag{20}$$

Finally, we average this inequality by the outcomes in A. The correlators are equal to

$$\begin{aligned}\sum_A P(A) J_i^A \langle J_i^B \rangle_A &= \sum_A P(A) J_i^A \sum_B P(B|A) J_i^B \\ &= \sum_{A,B} P(A,B) J_i^A J_i^B = \langle J_i^A J_i^B \rangle ,\end{aligned} \tag{21}$$

while $\sum_A P(A) J_A^B = \langle J^B \rangle \leqslant 1$. Thus

$$\left| \langle J_1^A J_2^B \rangle - \langle J_2^A J_2^B \rangle \right| \leqslant \sqrt{2}. \tag{22}$$

When the system is composed of two qubits, J^i is replaced with a corresponding Pauli operator and the inequality becomes

$$\mathcal{S}\left(\theta, \theta + \frac{\pi}{2}\right) = |\left\langle \hat{\sigma}_1^{(A)} \hat{\sigma}_1^{(B)} \right\rangle - \left\langle \hat{\sigma}_\perp^{(A)} \hat{\sigma}_\perp^{(B)} \right\rangle| \leqslant \sqrt{2} \tag{23}$$

for all systems compatible with the LHV model outlined above. To test it, one can analyse the combination of the correlator $\mathcal{B}$ in Main Eq. 6 and plotted in Main Fig. 4.

CHSH inequality for pair-scattering systems

The most general LHV theory that can be tested with two qubits assumes binary outcomes of the measurements in A and B. Both the original Bell inequality [1] or its modification proposed by Clauser, Horne, Shimony and Holt (CHSH) [2] use the above assumption. The CHSH inequality

$$\mathrm{B}_{\mathrm{CHSH}} = \left| E(\theta, \phi) + E(\theta', \phi') + E(\theta', \phi) - E(\theta, \phi') \right| \leqslant 2, \tag{24}$$

requires independent rotations in A and B with $\hat{R}_y^{(A)}(\theta) = \exp\left(-i\theta\hat{\sigma}_y^{(A)}\right)$ and $\hat{R}_y^{(B)}(\phi) = \exp\left(-i\phi\hat{\sigma}_y^{(B)}\right)$ and the measurement of the corresponding correlator

$$E(\theta, \phi) = \left\langle \hat{\sigma}_z^{(A)} \hat{\sigma}_z^{(B)} \right\rangle_{\theta,\phi}. \tag{25}$$

To derive the CHSH inequality for the system where atoms scatter in a pair, we use the bosonic field operators $\hat{\Psi}_\alpha(\mathbf{r})$ for each spin component $\alpha = \pm 1, 0$. The Hamiltonian (the summation convention is used) for a $J = 1$ BEC reads

$$\begin{aligned}\hat{H} = \int d^3r \Bigg[&\frac{\hbar^2}{2m} \nabla \hat{\Psi}_\alpha^\dagger(\mathbf{r}) \cdot \nabla \hat{\Psi}_\alpha(\mathbf{r}) + V(\mathbf{r}) \hat{\Psi}_\alpha^\dagger(\mathbf{r}) \hat{\Psi}_\alpha(\mathbf{r}) \\ &+ \frac{c_0}{2} \hat{\Psi}_\alpha^\dagger(\mathbf{r}) \hat{\Psi}_\beta^\dagger(\mathbf{r}) \hat{\Psi}_\beta(\mathbf{r}) \hat{\Psi}_\alpha(\mathbf{r}) \\ &+ \frac{c_1}{2} \hat{\Psi}_\alpha^\dagger(\mathbf{r}) \hat{\Psi}_\beta^\dagger(\mathbf{r}) \mathbf{F}_{\alpha\alpha'} \cdot \mathbf{F}_{\beta\beta'} \hat{\Psi}_{\beta'}(\mathbf{r}) \hat{\Psi}_{\alpha'}(\mathbf{r}) \Bigg].\end{aligned} \tag{26}$$

The coefficients $c_{0/1}$ are related to the scattering lengths $a_{0/2}$ in total angular momentum interaction channels $J = 0, 2$ by $c_0 = \frac{4\pi\hbar^2}{m} \frac{a_0 + 2a_2}{3}$ and $c_1 = \frac{4\pi\hbar^2}{m} \frac{a_2 - a_0}{3}$, while $\mathbf{F} = (F_x, F_y, F_z)$ is a vector of spin-1 matrices.

Though the scattering of $m_J = 0, 1$ pairs from BECs is governed by terms proportional to c_0 and c_1, in the low-density limit spin changing collisions are less probable, and the term proportional to c_1 can be safely neglected. Also in this regime, the dynamics of $\hat{\Psi}_\alpha$ can be found using the Bogoliubov approximation. Each component is decomposed into the dominant c-number term and a quantum correction, $\hat{\Psi}_\alpha(\mathbf{r}) = \phi_\alpha(\mathbf{r}) + \hat{\delta}_\alpha(\mathbf{r})$. The coherent fields ϕ_α are governed by time-dependent Gross-Pitaevskii-type equations, whereas the fields $\hat{\delta}_\alpha$ are subject to dynamical Bogoliubov equations which contain both $\hat{\delta}_\alpha$ and $\hat{\delta}^\dagger_\beta$ terms.

Since, in the process of the halo formation, the mean-field energy due to the BECs is small compared to the kinetic energy of the scattered atoms, the only relevant term proportional to c_0 is the production term, and the equation of motion is $i\hbar\partial_t\hat{\delta}_\alpha = -\frac{\hbar^2\nabla^2}{2m}\hat{\delta}_\alpha + c_0\phi_\beta\phi_\alpha\hat{\delta}^\dagger_\beta$. The presence of the field $\hat{\delta}^\dagger_\beta$ describes the scattering of atoms from BECs and the formation of the halo. The terms proportional to $\phi_0^2\hat{\delta}^\dagger_0$ and $\phi_1^2\hat{\delta}^\dagger_1$ govern the quantum depletion of the BECs, irrelevant for the scattering process. As a result, the Bogoliubov equations are symmetric for $\hat{\delta}_{0/1}$. Therefore, we can consider the same Bell sequence as in Ref. [3].

The Bell test starts with the mixing of the two spin components $m_J = 0, 1$ (from now on denoted as $\downarrow$ / $\uparrow$) independently in two opposite regions of the halo, A and B, by the angles ϕ and θ over the y-axis. The many-body angular momentum operators (and the atom-number operators) are

$$\hat{S}^\alpha_x = \frac{1}{2}\int_\alpha \frac{d\mathbf{k}}{2\pi}\left(\hat{\delta}^\dagger_\uparrow(\mathbf{k})\hat{\delta}_\downarrow(\mathbf{k}) + \hat{\delta}^\dagger_\downarrow(\mathbf{k})\hat{\delta}_\uparrow(\mathbf{k})\right), \tag{27}$$

$$\hat{S}^\alpha_y = \frac{1}{2i}\int_\alpha \frac{d\mathbf{k}}{2\pi}\left(\hat{\delta}^\dagger_\uparrow(\mathbf{k})\hat{\delta}_\downarrow(\mathbf{k}) - \hat{\delta}^\dagger_\downarrow(\mathbf{k})\hat{\delta}_\uparrow(\mathbf{k})\right), \tag{28}$$

$$\hat{S}^\alpha_z = \frac{1}{2}\int_\alpha \frac{d\mathbf{k}}{2\pi}\left(\hat{\delta}^\dagger_\uparrow(\mathbf{k})\hat{\delta}_\uparrow(\mathbf{k}) - \hat{\delta}^\dagger_\downarrow(\mathbf{k})\hat{\delta}_\downarrow(\mathbf{k})\right), \tag{29}$$

$$\hat{N}_\alpha = \frac{1}{2}\int_\alpha \frac{d\mathbf{k}}{2\pi}\left(\hat{\delta}^\dagger_\uparrow(\mathbf{k})\hat{\delta}_\uparrow(\mathbf{k}) + \hat{\delta}^\dagger_\downarrow(\mathbf{k})\hat{\delta}_\downarrow(\mathbf{k})\right), \tag{30}$$

with $\alpha = A, B$. Finally, a normalised correlator is constructed

$$E(\theta,\phi) = \frac{\langle \hat{S}^{(A)}_z \hat{S}^{(B)}_z\rangle_{\theta,\phi}}{\langle \hat{N}^{(A)}\hat{N}^{(B)}\rangle_{\theta,\phi}}, \tag{31}$$

where the subscript θ, ϕ denotes averaging over the rotated state.

This correlator $E(\theta, \phi)$ from Supplementary Eq. 31 satisfies the Bell inequality of Supplementary Eq. 24 for all LHV theories [4]. The correlator E can be evaluated analytically in the Bogoliubov theory [3]: $E(\theta,\phi) = -\mathcal{E}\cos(\theta+\phi)$, showing a dependence only in the sum of the angles and oscillations with the amplitude $\mathcal{E} = (g^{(2)}_{\uparrow\downarrow} - 1)/(g^{(2)}_{\uparrow\downarrow} + 1)$, which is expressed in terms of the two-particle correlation function:

$$g^{(2)}_{\uparrow\downarrow} = \frac{\iint\limits_{A\,B} d\mathbf{k}d\mathbf{k}'\left\langle \hat{\delta}^\dagger_\uparrow(\mathbf{k})\hat{\delta}^\dagger_\downarrow(\mathbf{k}')\hat{\delta}_\downarrow(\mathbf{k}')\hat{\delta}_\uparrow(\mathbf{k})\right\rangle}{\iint\limits_{A\,B} d\mathbf{k}d\mathbf{k}'\left\langle \hat{\delta}^\dagger_\uparrow(\mathbf{k})\hat{\delta}_\uparrow(\mathbf{k})\right\rangle\left\langle \hat{\delta}^\dagger_\downarrow(\mathbf{k}')\hat{\delta}_\downarrow(\mathbf{k}')\right\rangle}. \tag{32}$$

Here, the average is calculated in the state prior to rotations. The expression for $\mathrm{B_{CHSH}}$ optimised over angle settings yields the condition $|\mathcal{E}| > 1/\sqrt{2}$, which in turn is equivalent to $g^{(2)}_{\uparrow\downarrow} > 2\sqrt{2} + 3$ for the Bell inequality (Supplementary Eq. 24) to be violated.

The key quantities in the Bell inequality (Supplementary Eq. 24) are probed by setting equal angles in the correlator, i.e., $E(\theta,\theta) = \mathcal{B}(\theta)$. The observation of $|\mathcal{B}(\theta)| > 1/\sqrt{2}$ signals the detection of the Bell correlations, and the potential for the Bell inequality violation in an actual Bell test experiment with independent settings of the angles in separated regions A and B. Finally, we point out that in the low-gain regime, when only a single pair of qubits is scattered, the many-body angular momentum operators are replaced by the Pauli matrices in A and B, and the correlator (Supplementary Eq. 31) takes the form of Supplementary Eq. 25.

Two-body correlator $\mathcal{B}$

The correlation coefficient $E(\theta, \phi)$ given above can be readily evaluated from the single-particle detection resolved in momentum and spin, since

$$E(\theta, \phi) = \frac{\sum_{\mathbf{k}\in V} \langle \hat{S}_z^{(\mathbf{k})} \hat{S}_z^{(-\mathbf{k})} \rangle_{\theta,\phi}}{\sum_{\mathbf{k}\in V} \langle \hat{N}^{(\mathbf{k})} \hat{N}^{(-\mathbf{k})} \rangle_{\theta,\phi}} = \frac{\left\langle \left(\hat{N}_\uparrow^{(A)} - \hat{N}_\downarrow^{(A)}\right)\left(\hat{N}_\uparrow^{(B)} - \hat{N}_\downarrow^{(B)}\right)\right\rangle_{\theta,\phi}}{\left\langle \left(\hat{N}_\uparrow^{(A)} + \hat{N}_\downarrow^{(A)}\right)\left(\hat{N}_\uparrow^{(B)} + \hat{N}_\downarrow^{(B)}\right)\right\rangle_{\theta,\phi}}. \tag{33}$$

Therefore, it can be expanded in terms of products of number operators across the regions A/B

$$E = \frac{\left\langle \hat{N}_\uparrow^{(A)} \hat{N}_\uparrow^{(B)} \right\rangle + \left\langle \hat{N}_\downarrow^{(A)} \hat{N}_\downarrow^{(B)} \right\rangle - \left\langle \hat{N}_\uparrow^{(A)} \hat{N}_\downarrow^{(B)} \right\rangle - \left\langle \hat{N}_\downarrow^{(A)} \hat{N}_\uparrow^{(B)} \right\rangle}{\left\langle \hat{N}_\uparrow^{(A)} \hat{N}_\uparrow^{(B)} \right\rangle + \left\langle \hat{N}_\downarrow^{(A)} \hat{N}_\downarrow^{(B)} \right\rangle + \left\langle \hat{N}_\uparrow^{(A)} \hat{N}_\downarrow^{(B)} \right\rangle + \left\langle \hat{N}_\downarrow^{(A)} \hat{N}_\uparrow^{(B)} \right\rangle}, \tag{34}$$

where for convenience the subscript (θ, ϕ) for labelling the general rotated state is assumed for all correlators. Since the density of the scattering halo is symmetric in momentum (s-wave scattering) and spin, $\bar{N} = \left\langle \hat{N}_m^{(\mathbf{k})} \right\rangle$ for all $m \in \{\uparrow, \downarrow\}$ and $\mathbf{k} \in V$, observe that each term in Supplementary Eq. 34 corresponds to a second-order correlation function

$$\left\langle \hat{N}_i^{(A)} \hat{N}_j^{(B)} \right\rangle = \bar{N}^2 g_{ij}^{(2)} \tag{35}$$

Therefore, the general correlation coefficient $E(\theta, \phi)$ in Supplementary Eq. 34 can be written in terms of $g^{(2)}$ as

$$E(\theta, \phi) = \frac{g_{\uparrow\uparrow}^{(2)} + g_{\downarrow\downarrow}^{(2)} - g_{\uparrow\downarrow}^{(2)} - g_{\downarrow\uparrow}^{(2)}}{g_{\uparrow\uparrow}^{(2)} + g_{\downarrow\downarrow}^{(2)} + g_{\uparrow\downarrow}^{(2)} + g_{\downarrow\uparrow}^{(2)}}. \tag{36}$$

SUPPLEMENTARY NOTE 2: CHARACTERISATION OF SPIN ROTATION

Rabi oscillation

This section provides the experimental details used to obtain the Rabi oscillation in Main Fig. 3, from which we concluded that the experimental operation in Main Fig. 1(b) corresponds to a coherent rotation of the qubit. The characterisation follows a sequence almost identical to the main experiment (see Main Fig. 1), except that only $m_J = +1$ states are prepared initially. Here the collision pulse is applied at $t \approx 0$ so that a Bragg transition (no change in internal state) can be driven with the same beam geometry used to produce the entangled pairs (see Main Fig. 1(a)), producing a $|\uparrow\rangle$-polarised scattering halo (see left Supplementary Figure 1(a)). At the time and location of the Bragg collision pulse, the magnetic field points along the x-axis similar to [5], after which it is stabilised to $\mathbf{B}_0$ for the rotation pulse as described in the previous section. The rotation pulse is applied at $t = 3.8$ ms so that the halo is $d_{\text{sep}} \approx 0.46$ mm in diameter, approximately five times larger than the $|\Psi^+\rangle$-halo at the point of rotation, but still significantly smaller than the beam waist.

The resulting atom number density at the detector is shown in Supplementary Figure 1(a) for various durations of rotation pulse τ. The total number of atoms detected in the truncated scattering volume (details are given in the next section) N'_α is shown in Supplementary Figure 1(b) for various rotation pulse durations. By defining the population fraction of the internal state $\alpha = \pm 1, 0$, by $P(\alpha) = N'_\alpha / N'$, where $N' = \sum_\alpha N'_\alpha$ is the sum over the triplet, we obtain the Rabi oscillation shown in Main Fig. 3. Although negligible transfer to $m_J = -1$ was achieved, we noticed a loss in the total number of atoms detected in the scattering volume, $N'_{\text{loss}}(\tau) = N'(0) - N'(\tau)$ (see Supplementary Figure 1(b)). Two key features evident in the behaviour of the total atom number during the Raman transition are the loss strongly correlated with the presence of $m_J = 0$ states and the steady, but weak increase of the total population, which we explain below.

The significant decrease in the total atom number, observed to be up to 25% and correlated with the N'_0 population (see Supplementary Figure 1(b)), is due to Penning ionisation between pairs of He* atoms [6], which is enhanced by 4 orders of magnitude for spin unpolarised pairs, compared to pairs of $m_J = +1$ atoms.

In our experiment, Penning ionisation would most strongly affect the entangled pairs at the earliest time following their production, since the local density of atoms is the highest during the spatial overlap of the pairs with the BECs, allowing for

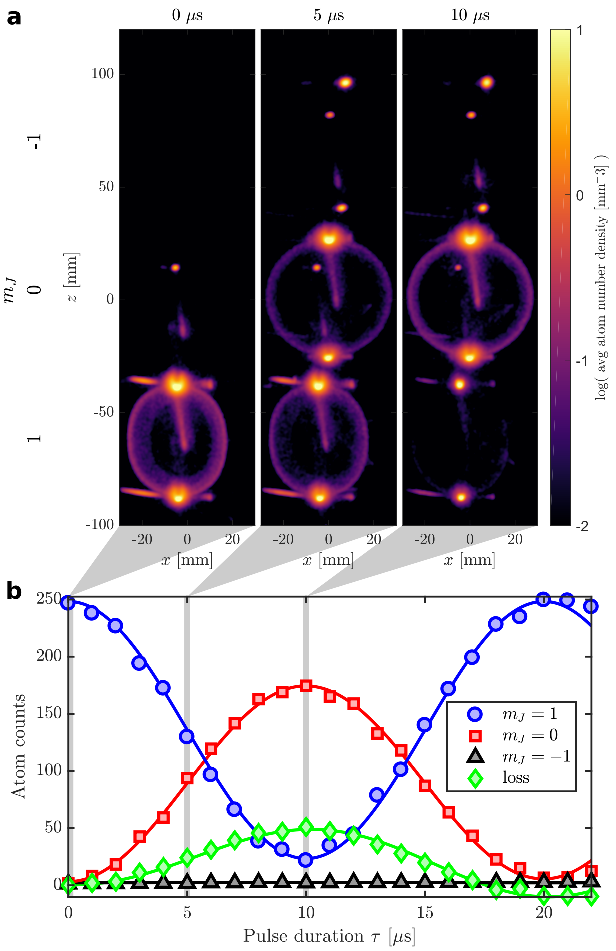

Supplementary Figure 1. Characterisation of the rotation pulse. (a) Atom count density at the detector in the zx-plane, integrated over -12 mm $< y <$ 15 mm, for different spin rotation pulses applied to $|\uparrow\rangle$-polarised scattering halo. (b) Atom counts in each scattering halo. Significant loss in total number is observed (green diamond), which is most likely due to Penning ionisation based on the strong correlation with population in $m_J = 0$. For longer pulse durations the atom number in the scattering volume increases due to single-photon absorption of the Raman beams by the BECs. Solid lines are fits to data, and statistical uncertainties (namely the standard error in the mean) are smaller than the graphical markers.

more frequent Penning ionising collisions. After the BEC and entangled pair wavefunctions have spatially separated, there should be a negligible fraction of atoms lost, since the $|\Psi^+\rangle$ pair source was prepared with very low numbers - an order of in magnitude lower than the scattering halo used to characterise the Raman pulse in Supplementary Figure 1. In the presence of BECs in both spin-states, each atom from the $|\Psi^+\rangle$ pair is almost equally likely to be lost by Penning ionisation. The loss of an atom from any pair results in the detection of a single-hit event, i.e. a single-hit event will be detected in A with $\uparrow$ or $\downarrow$, with no correlated hit in B. Such events are thus naturally treated by the correlation functions $g^{(2)}_{ij}$ as an uncorrelated background event (see Main Eq. 3). The presence of Penning ionisation will therefore strictly can only reduce the observed correlator to the asymptotic uncorrelated state $\mathcal{B} = 0$, such that in the extreme case where half of every pair is lost, $g^{(2)}_{ij} = 1$ for all back-to-back correlations will be observed.

The increase in total number ($N'_{\text{loss}} < 0$) for longer pulse durations ($\tau > 17$ μs) is due to the constant single-photon absorption of the Raman beams and subsequent decay by the BECs ($2\,^3\mathrm{S}_1 \to 2\,^3\mathrm{P}_J \to 2\,^3\mathrm{S}_1$ where each process is accompanied with a single photon recoil), into a scattering volume in momentum space intersecting the s-wave scattering halo. The entangled atoms of interest originally occupying the halos are indeed equally subject to such lossy processes, and contribute to an additional loss term, negligible in comparison to that of BECs due to the relative atom numbers involved. In our experiment, a large detuning of the Raman beams from resonance minimised the rate of single-photon absorption, as demonstrated by the nearly absent scattering effects even from the BECs.

Ramsey fringes

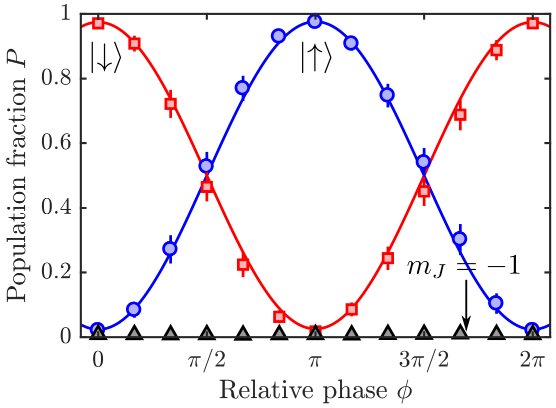

Supplementary Figure 2. Ramsey-type fringe from two separated $\pi/2$-pulses with a phase delay in the second rotation pulse. The large fringe visibility of $0.95(2)$ demonstrates the ability to independently configure the axis of rotation ($\mathbf{n}$) around the xy-plane of the Bloch sphere. Error bars indicate one standard error in the mean.

As shown in Main Fig. 3, a Rabi oscillation through 2π-rotation can be induced between the $|\uparrow\rangle$ and $|\downarrow\rangle$ states, with negligible coupling to the $m_J = -1$ state. To ensure that the Raman pulse is producing the desired rotation of the atomic spin on the Bloch sphere, we implement a Ramsey-type interferometry where a secondary $\pi/2$-pulse with a phase delay ϕ follows the first $\pi/2$-pulse $\hat{R}_y(\pi/2)$. Following an identical initialisation scheme to the Rabi oscillation experiment, the fixed delay between the two $\pi/2$-pulses was set at $T \approx 10 \cdot T_{\mathrm{L}}$, and the phase delay between the two Raman optical fields scanned over all range between 0 and 2π, relative to the $\hat{R}_y$ reference pulse. We observe a Ramsey-type fringe with a high visibility of $0.95(2)$ in Supplementary Figure 2 which along with the Rabi oscillation (see Main Fig. 3) provides a clear demonstration of the desired unitary rotations on the atomic spin $\hat{R}_\phi(\theta) = \exp(-i\theta(\cos\phi\,\hat{\sigma_x} + \sin\phi\,\hat{\sigma_y})/2)$.

SUPPLEMENTARY NOTE 3: CORRELATION FUNCTIONS

This section provides details on the two-particle correlation functions in the scattering halos which were ultimately used to construct the $\mathcal{B}$ correlator. Supplementary Figure 3(a) shows the effect of the rotation sequence on the correlation properties of the initially spin and momentum anti-correlated pairs by second-order correlation functions $g^{(2)}$ as defined in Main Eq. 3. As expected for the $|\Psi^+\rangle$ scattered pairs ($\tau = 0\ \mu$s) no correlation is seen between oppositely scattered pairs with the same spin, however with a common $\pi/2$ rotation ($\tau = 5\ \mu$s) the spins are always parallel, and finally return to anti-parallel by the nearly spin-flipping π pulse ($\tau = 10\ \mu$s). The asymmetry in the Gaussian correlation profiles between different spin types are due to the effects from stray magnetic field during the atoms' free-fall before detection (see previous section for details) which distorts the atomic velocities and thus the correlation profile. The difference in signal-to-noise ratios of the correlation functions for different rotation sequences are due to the variations in amount of acquired data.

The relative strengths in spin correlations with various rotation pulse durations are summarised in Supplementary Figure 3(b). Here, the normalised second-order correlation is defined as

$$\mathcal{G}_{ij} = \frac{2 g^{(2)}_{ij}}{\sum_{\alpha,\beta \in \{\uparrow,\downarrow\}} g^{(2)}_{\alpha\beta}},$$

where the second-order correlation without any argument denotes the value at the exact back-to-back (BB) condition in momentum ($g^{(2)}_{ij} = g^{(2)}_{ij}(\Delta\mathbf{k} \approx 0)$). The BB correlation strengths were determined from a cubic bin of width 0.0138 in the normalised momentum unit. As demonstrated in the theory section, the general bipartite correlator can be determined directly from the normalised correlations by Supplementary Eq. 36. The observed asymmetry in the correlation strengths between $\uparrow\uparrow$ and $\downarrow\downarrow$ combinations can be explained by the asymmetry in the correlation volumes due to the shape distortion of the $m_J = 1$ scattering halo, such that the correlation volume integrated strengths recover the expected symmetry.

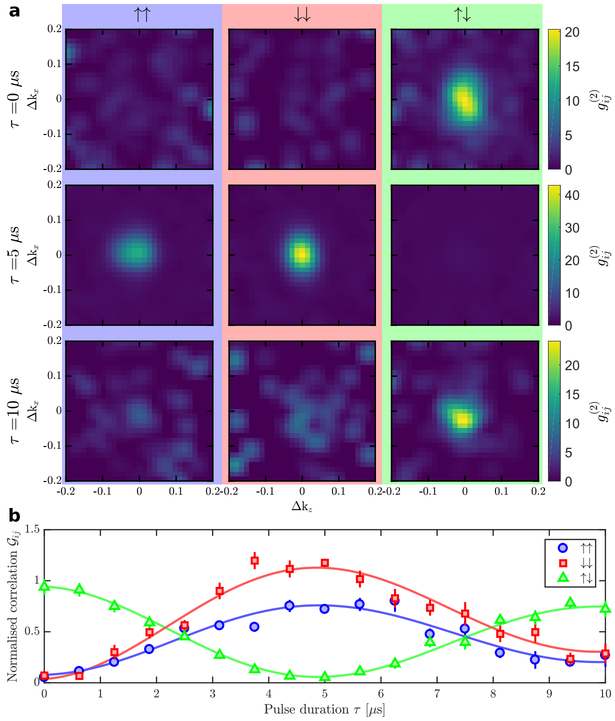

Supplementary Figure 3. Second-order correlation functions after spin rotations. (a) $\Delta k_y = 0$ slices of $g^{(2)}(\Delta \mathbf{k})$ for various spin rotation pulses (separated by rows) and spin-pairing configurations (separated by columns). (b) Normalised correlations for the back-to-back condition ($\Delta \mathbf{k} = 0$). The asymmetry in correlation between ↑↑ (blue circles) and ↓↓ (red squares) arises from the shape distortion of $|\uparrow\rangle$ atoms' trajectories by stray magnetic fields during time-of-flight. Error bars correspond to the standard error estimated from bootstrapping. Solid lines are damped sine curve fits to data.

Error analysis

All statistical uncertainties in the correlation functions, and similarly for other variables, were determined from bootstrapping described here. From the complete dataset from which the representative correlation function g is determined (see Main Eq. 3), subsets (fractional size η) are sampled with replacement and analysed identically to produce a distribution of outcomes G. A robust estimate of the standard error of the mean (correlation function in this case) is then given by $\sigma_{\bar{g}} = \sqrt{\eta \cdot \mathrm{Var}(G)}$, independent of the sampling size.

SUPPLEMENTARY REFERENCES

[1] Bell, J. S. On the Einstein Podolsky Rosen paradox. *Physics* **1**, 196–200 (1964).

[2] Clauser, J. F., Horne, M. A., Shimony, A. & Holt, R. A. Proposed experiment to test local hidden-variable theories. *Phys. Rev. Lett.* **23**, 880–884 (1969).

[3] Wasak, T. & Chwedeńczuk, J. Bell inequality, Einstein-Podolsky-Rosen steering, and quantum metrology with spinor Bose-Einstein condensates. *Phys. Rev. Lett.* **120**, 140406 (2018).

[4] Reid, M. D. & Walls, D. F. Violations of classical inequalities in quantum optics. *Phys. Rev. A* **34**, 1260–1276 (1986).

[5] Khakimov, R. I. *et al.* Ghost imaging with atoms. *Nature* **540**, 100–103 (2016).

[6] Vassen, W. *et al.* Cold and trapped metastable noble gases. *Rev. Mod. Phys.* **84**, 175–210 (2012).
